# Green and Cost-Effective Synthesis of Metallic Nanoparticles by Algae: Safe Methods for Translational Medicine

**DOI:** 10.3390/bioengineering7040129

**Published:** 2020-10-16

**Authors:** Bushra Uzair, Ayesha Liaqat, Haroon Iqbal, Bouzid Menaa, Anam Razzaq, Gobika Thiripuranathar, Nosheen Fatima Rana, Farid Menaa

**Affiliations:** 1 Department of Bioinformatics and Biotechnology, Islamic International University, Islamabad 44000, Pakistan; bushra.uzair@iiu.edu.pk (B.U.); ayesha.bsbio23@iiu.edu.pk (A.L.); 2 College of Pharmaceutical Sciences, Soochow University, Suzhou 215123, China; harooniqbal415@hotmail.com (H.I.); anamrazzaq.ajk@gmail.com (A.R.); 3 Department of Oncology and Nanomedicine, California Innovations Corp., San Diego, La Jolla, CA 92037, USA; bouzid.menaa@gmail.com; 4 Institute of Chemistry Ceylon, College of Chemical Sciences, Welikada, Rajagiriya 10107, Sri Lanka; tgobika@ichemc.edu.lk; 5 Department of Biomedical Engineering & Sciences, School of Mechanical & Manufacturing Engineering, National University of Sciences & Technology, Islamabad 44000, Pakistan; nosheen.fatima@smme.nust.edu.pk

**Keywords:** algal nanotechnology, nanoparticle biosynthesis, metallic nanoparticles, nanomedicine, translational medicine, sustainable technology

## Abstract

Metal nanoparticles (NPs) have received much attention for potential applications in medicine (mainly in oncology, radiology and infectiology), due to their intriguing chemical, electronical, catalytical, and optical properties such as surface plasmon resonance (SPR) effect. They also offer ease in controlled synthesis and surface modification (e.g., tailored properties conferred by capping/protecting agents including N-, P-, COOH-, SH-containing molecules and polymers such as thiol, disulfide, ammonium, amine, and multidentate carboxylate), which allows (i) tuning their size and shape (e.g., star-shaped and/or branched) (ii) improving their stability, monodispersity, chemical miscibility, and activity, (iii) avoiding their aggregation and oxidation over time, (iv) increasing their yield and purity. The bottom-up approach, where the metal ions are reduced in the NPs grown in the presence of capping ligands, has been widely used compared to the top-down approach. Besides the physical and chemical synthesis methods, the biological method is gaining much consideration. Indeed, several drawbacks have been reported for the synthesis of NPs via physical (e.g., irradiation, ultrasonication) and chemical (e.g., electrochemisty, reduction by chemicals such as trisodium citrate or ascorbic acid) methods (e.g., cost, and/ortoxicity due to use of hazardous solvents, low production rate, use of huge amount of energy). However, (organic or inorganic) eco-friendly NPs synthesis exhibits a sustainable, safe, and economical solution. Thereby, a relatively new trend for fast and valuable NPs synthesis from (live or dead) algae (i.e., microalgae, macroalgae and cyanobacteria) has been observed, especially because of its massive presence on the Earth’s crust and their unique properties (e.g., capacity to accumulate and reduce metallic ions, fast propagation). This article discusses the algal-mediated synthesis methods (either intracellularly or extracellularly) of inorganic NPs with special emphasis on the noblest metals, i.e., silver (Ag)- and gold (Au)-derived NPs. The key factors (e.g., pH, temperature, reaction time) that affect their biosynthesis process, stability, size, and shape are highlighted. Eventually, underlying molecular mechanisms, nanotoxicity and examples of major biomedical applications of these algal-derived NPs are presented.

## 1. Introduction

Nanotechnology is a vibrant, revolutionary, and fast-developing multidisciplinary field that deals at nanoscopic scale (10^−9^ m), impacting the environment, health, and socio-economy for more than two decades [1,2,3,4]. The resulting (natural, engineered or incidental) nanomaterials (e.g., nanoparticles (NPs)) usually display a dimension below 100 nm along with unique physical–chemical–biological properties (e.g., large surface-to-volume ratio, surface functionalization, controlled targeting and release) compared to their counterpart bulk materials. These interesting features make them incredibly attractive tools for applications in various fields (e.g., cosmetology, pharmacy, biotechnology, chemistry, or agriculture).

Actually, metal and non-metal NPs synthesized from plants (e.g., seaweeds) and microorganisms (e.g., bacteria) have attained more attention than classical physical and chemical synthesis routes mainly because of their (i) green assembly during the biosynthesis that uses clean energy processes regulated naturally, which subsequently overcomes the health and environmental toxicity; (ii) various attributes that contrast with those of them in the massive structure; (iii) peerless characteristics that drastically depend on the type (e.g., morphology/shape, size distribution, composition, and stability) and the dose, which can both impact on toxicity; (iv) cost-effectiveness; (v) eligibility for usage in a wide range of activities encompassing cosmetics, theranostics, food and textile fields [1,2,3,4].

Importantly, a recent trend of biologically synthesizing NPs using algal species (e.g., *Chlorophyceae* (green), *Phaeophyceae* (brown), *Cyanophyceae* (blue green), *Rhodophyceae* (red), Diatoms) is developing [5]. This tremendous and increasing interest is mainly due to the (i) ease of handling algae; (ii) capacity of algae to absorb/accumulate inorganic metallic ions; (iii) alternative strategy for cost-effective synthesis; and (iv) naturally eco-friendly, fast and healthier synthesis methods [5].

Algae represent an autotrophic (i.e., producers of their own food using light, water, carbon dioxide, or other chemicals) and polyphyletic (i.e., large and diverse) group of photosynthetic eukaryotic organisms. They are classified as microalgae (unicellular such as diatoms or multicellular) and macroalgae (seaweeds), primarily based on their morphological features, and can be found in marine and freshwater environments or over moist rocks [6]. These aquatic plants are simple and non-flowering. Indeed, although algae contain chlorophyll, they are lacking various structures (e.g., true stems, roots, leaves, and vascular tissue) that characterize the land plants (e.g., bryophytes, tracheophytes) [7]. Even so, they play a key role in the aquatic ecosystems (outside of their possible toxic blooming, which, interestingly, can be controlled by algal-mediated NPs) and represent an economically valuable biomass source for various applications (e.g., agricultural, aquacultural, pharmaceutical, cosmetical, biotechnological, energetical, and nanotechnological) [8,9].

This article mainly reports key factors and methods used in the algal-mediated synthesis of NPs. It also highlights (potential) biomedical applications, underlying molecular mechanisms and nanotoxicity of algal-mediated synthetized metallic NPs, with a special emphasis on AgNPs and AuNPs. Eventually, this review aims to contribute to the development of safe and sustainable nanotechnology from aquatic ecosystems.

## 2. Types of Nanoparticles and Applications

There are two kinds of NPs that can be synthesized using algae: the organic and inorganic ones.

Organic NPs include poly-ε-lysine (ε-PL), chitosan (CS), cationic quaternary polyelectrolytes and quaternary ammonium compounds. Generally, these organic NPs are weakly stable at high temperatures compared to that of inorganic NPs, favoring inorganic NPs as antimicrobial polymers [10]. Even so, **ε**-PL is a L-lysine cationic homopeptide which is very efficient against Gram-positive bacteria and spores of *B. coagulans*, *B. subtilis*, and *B. stearothermophilus* [11,12]. CSNPs possess a wide spectrum of antimicrobial activity against viruses, bacteria, and fungi. They are nontoxic, biocompatible and can serve as absorption enhancers [13,14]. Cationic quaternary polyelectrolytes represent by-products of acrylic and methacrylic compounds. Due to their structural versatility, these molecules have a broad variety of biological applications (e.g., as antimicrobials) when factors such as their hydrophobicity, surface charge, molecular weight are altered [15]. Quaternary ammonium compounds are eminent disinfectants and rely on chain length for their antimicrobial properties [16]. Interestingly, the synthesis of organic NPs by algae has been poorly studied so far; This may be due to the lack of stability of the organic NPs at high temperature. Recently, Tiburu et al. (2017) investigated the formation of CSNPs by isolating deacetylated chitin from freshwater green algae [17]. The chitin was then converted into CS by mixing it with caustic soda and heating it by indirect steam. The resulting CSNPs were of an orthorhombic structure as revealed by x-ray powder diffraction (XRD) analysis.

Inorganic NPs, which include Ag, zinc oxide (ZnO), copper oxide (CuO), Au, and iron oxide (magnetite Fe_3_O_4_ and/or its oxidized form maghemite γ-Fe_2_O_3_), are the focus of this article. Metal NPs are the most studied nanomaterials because they display unique catalytic, electronic, and optical properties [18]. They also offer ease in controlled synthesis and surface modification, which allows tuning their size and shape-dependent properties [18]. It is now established that AgNPs possess strong antimicrobial properties against many fungi, viruses and bacteria because of their action as a photocatalyst and the capability to produce reactive oxygen species (ROS) [19,20,21,22]. ZnONPs are low cost antibacterial agents that can restrict the growth of a wide array of pathogenic bacteria [23]. They show anti-biofilm activity and can also block UV-rays, thus representing an effective coating material in medical and food materials [24]. CuONPs are used as antibacterial agents but they are less efficient than AgNPs or ZnONPs [25]. Their antibacterial activity is exerted through their capacity to disrupt cell membranes and produce ROS [26]. AuNPs are considered as non-toxic and are mainly used for gene delivery applications, biosensing and cancer therapy [27,28,29]. Iron oxide NPs are much applied in magnetic resonance imaging (MRI), immunoassays, tissue repair, and as effective agents in chemotherapy [30,31].

## 3. General Methods for Synthesizing Nanoparticles with Tailored Properties

NPs synthesis can follow two different pathways by using either the technique "bottom-up" or by applying the technique “top-down”. The “bottom-up” technique is also called the self-assembly method because the initial synthesized NPs combine to form a structure or cluster by using chemical or biological methods. In “top-down” technique (e.g., ion etching, lithography), an appropriate bulk content is reduced to fragments using either chemical or physical processes. 

NPs obtained from the “bottom-up” method (e.g., chemical/physical vapor deposition, electrochemistry) are preferred since they display more homogenous chemical composition and lesser defects than those resulting from “top-down” methods that elicit imperfect surface structures. These defected surface structures can have harmful impacts on the physical and chemical properties of NPs [32].

Wet-chemical processes are commonly used methods for synthesizing NPs, and implicate that NPs are grown in liquid media in the presence of several reactants, reducing and stabilizing/protecting agents (e.g., capping agents/ligands). Typically, NPs are synthesized by chemical reduction either by organic or inorganic reducing agents, via the colloidal route or by sol-gel method [32,33,34]. Although these methods yield high production of NPs at a low cost, they also contain some drawbacks (e.g., use of hazardous solvents, processing of harmful by-products and contamination from precursor chemicals) [32].

Physical synthesis of NPs involves various methods such as ultrasonication, electron beam, ion implantation, laser radiation, spray pyrolysis and vapor phase [34]. However, these physical methods have some drawbacks that make them less suitable for synthesizing NPs (e.g., high cost, low production rate, use of a huge amount of energy for maintaining the high temperature and pressure) [32].

To eliminate the drawbacks of physical and chemical methods used in the synthesis of NPs, an increasing interest is observed for green NPs synthesis using biological entities (e.g., microorganisms including bacteria, fungi, and yeast, microalgae or plant extracts including from macroalgae), which are considered as safe for health while minimizing the scarcity of energy resources [5,32].

Eventually, the choice of preparation procedure not only shall depend on the physical and chemical characteristics required on the final product (e.g., size (usually < 100 nm), dispersion, shape (e.g., spherical, star-shaped, or branched NPs), chemical miscibility, optical properties (surface plasmon resonance (SPR) effect), but also shall consider environmental aspects.

## 4. Key Factors Governing Nanoparticles Synthesis 

Several chemical, physical, and biological controlling factors are responsible for the proper, efficient, and optimal NPs (bio)synthesis process. These include pH, temperature, metal ion concentrations, reactant concentrations, reaction time, stirring rate, incubation time, capping agents, and the type of microorganism or plant extract used. Most of these factors, if not all, can have an impact on the stability, size, and shape of the NPs. Besides, it is well known that the toxicity of nanomaterials essentially depends on the structural features such as the size, shape, composition, and surface chemistry.

### 4.1. pH

During the NPs synthesis reaction, the buffer strength (i.e., pH) must be stable to avoid varying shapes and sizes of NPs [35]. At low pH, the SPR peak becomes wide and deflects towards a longer wavelength region which results in a wide range of NPs (e.g., mainly cylindrical or triangular shaped), whereas for synthesizing small-sized NPs, high pH is suitable which aids the formation of spherical NPs [5]. Thereby, Ghaemi et al. (2017) observed the effect of pH by varying it from 2 to 10 during the formation of AgNPs when the extract of the brown macroalgae, *Sargassum angustifolium* (C.Agardh) was used [36]. Interestingly, these NPs displayed different stabilization power according to acidic or alkaline microenvironments. Indeed, in alkaline conditions (e.g., pH 10), a reasonable number of very stable small-sized NPs were formed compared to that of in acidic conditions. In a more recent study, these observations were partially confirmed by Shou et al. (2011) that illustrated the formation of AuNPs in acidic and alkaline conditions by using triblock copolymers of PEO–PPO–PEO in aqueous media. Under alkaline conditions, many large-pearl-sized NPs were formed and were much more stable than the clustered NPs formed in acidic conditions [37,38].

### 4.2. Temperature

Temperature is another critical factor that governs the synthesis of NPs. The reaction temperature highly influences various chemical methods (e.g., solvothermal, electrochemical or templating methods) [39]. Generally, the NPs synthesis through green methodologies require a temperature of almost 100 °C [40]. Temperature higher than 350 °C is required for physical methods whereas low temperatures are required for chemical synthesis [5,41]. Studies showed that high rate of AuNPs formation was observed at high temperature, which could be explained by a faster reduction rate [36]. However, their average size decreased when their conversion rate increased [35].

### 4.3. Reactant Concentration

Varying concentrations of biomolecules in a plant extract can have a different influence on NPs formation [35]. Thereby, Chandran et al. (2006) investigated the effect of reducing agent concentration in the reaction mixture on the yield and size of Au nanotriangles synthetized by *Aloe vera* (L.) leaf extract used as the reducing agent [42]. Monitoring the formation of Au nanotriangles as a function of time using transmission electron microscopy (TEM) reveals that multiply twinned particles (MTPs) play an important role in the formation of Au nanotriangles. It was also observed that the slow rate of the reaction along with the shape directing effect of the constituents of the extract are responsible for the formation of single crystalline Au nanotriangles. 

Furthermore, it was observed that when extract concentrations (up to 20%) of the marine green macroalga *Caulerpa serrulata* (Forsskål) were added to a constant concentration of silver nitrate (AgNO_3_) solution at room temperature, the SPR band intensity was increased while a decrease in average size of AgNPs was obtained [43]. However, a further increase in the extract concentration (from 20% to 25%) reduced the SPR band intensity, but this effect was explained by a possible particle agglomeration.

### 4.4. Reaction Time

Reaction time is a crucial factor in the synthesis of NPs. Indeed, performing the same experiment by changing the reaction time may result in different particle sizes. For instance, Ahmad et al. (2012) biosynthesized the NPs by using *Ananas comosus* (L.) extract, which started to appear in 2 min and when the process was extended for 5 min, spherical NPs were formed with a mean size of 12 nm [44]. Additionally, Aboelfetoh et al. (2017) showed that the gradual increase in contact time/interaction between *Caulerpa serrulata* (Forsskål) and silver ion (Ag^+^) at room temperature leads to an increase in SPR peak intensity and a rapid synthesis of non-agglomerated AgNPs [43]. Moreover, Prathna et al. (2011) investigated the growth kinetics of AgNPs as synthesized on a reduction of silver nitrate (AgNO_3_) solution by aqueous extract of *Azadirachta indica* (Neem tree) leaves [45]. The reaction was continued for up to 4 h and analyzed by Ultraviolet-Visible (UV-Vis) spectrometry. The first peak of UV-Vis spectrum appeared after the first 2 h revealing NPs with particle sizes ranging from 10 to 35 nm. The XRD pattern obtained after 2 h showed that the NPs were spherical with particle sizes around 20 nm. As the time proceeds at almost 4 h, the micrograph showed NPs with an average size of 36.6 nm.

### 4.5. Capping Agent

As briefly mentioned earlier, basic procedures used for the preparation of protected or unprotected metallic NPs include colloidal chemistry methods (e.g., AuNPs synthesis by reduction with trisodium citrate, ascorbic acid, or sugars in aqueous phase; NPs passivation with alkanethiols, cetyltrimethylammonium bromide, or bovine serum albumin), as well as techniques such as microwave-assisted synthesis, NPs synthesis in ethylene glycol, and template-assisted synthesis with dendrimers [46]. The bottom-up approach where the metal ions are reduced by the reducing agent and the NPs grown in the presence of capping ligands has been widely used [18].

The biggest obstacle of using metal NPs could be their tendency to aggregate over time, which usually leads to a deterioration in their overall activity [18]. Thus, the stability of metal NPs against aggregation and oxidation should be adequately enhanced, including by using organic protecting ligands for which the head group interacts with a strong affinity to metal NP surfaces to stabilize the highly reactive surface atoms [18]. It is postulated that the alkyl spacer between the head and the tail groups of the ligand provides a capping shell and controls the interparticle spacing [18]. In addition, the functional tail groups of the ligand play a crucial role in determining the surface reactivity and solubility of the NPs [18]. Importantly, it was found that the type of ligands (e.g., thiol, disulfide, ammonium, citrate) and the degree of ligand capping along with applied synthetic conditions could systematically alter the NPs size, NPs shape, ligand–metal ratio and directly influence the chemical and physical properties (e.g., optical and electronic) of metal NPs [18].

### 4.6. Choice of the Organism 

According to Rai et al. (2013), cost-effective NPs biosynthesis depends not only on the above physical–chemical parameters but also on (i) the selection of the best organism according to important intrinsic properties such as the growth rate, biochemical pathways and enzyme activities; (ii) its inoculum size; (iii) the choice of biocatalysts, which is crucial to accelerate the rate of reaction (i.e., reduction), although this should be done carefully [47]. Biocatalysts may be used as whole cells, crude, or purified enzymes (e.g., NADH, NADPH, FAD). In this regard, living whole cells are preferred because these coenzymes are expensive and can be recycled during the pathway, besides proving their substantial effectiveness. 

## 5. Algal-Mediated Inorganic Nanoparticles Synthesis Methods

As previously evoked, algae are rich in polymeric molecules (e.g., polysaccharides) and can hyperaccumulate heavy metal ions and remodel them into malleable forms by (bio) reduction process. Algal extracts typically consist of pigments (e.g., chlorophylls, carotenoids, phycobilins), carbohydrates, proteins, minerals, polyunsaturated fatty acids (PUFAs), and other bioactive compounds such as antioxidants (e.g., polyphenols and tocopherols) that may be used as stabilizing/capping and reducing agents [48]. Moreover, the phycosynthesis of NPs takes less time compared to that of their biosynthetic entities [49]. Due to these overall properties, live or dead algae are used as a model organism for the eco-friendly synthesis procedure of bionanomaterials, such as metallic NPs [50]. In this routine, the NPs formation is followed by UV-Vis absorption spectroscopy and TEM, while the functional groups involved in the bioreduction are studied by FTIR [50].

**Figure 1:**
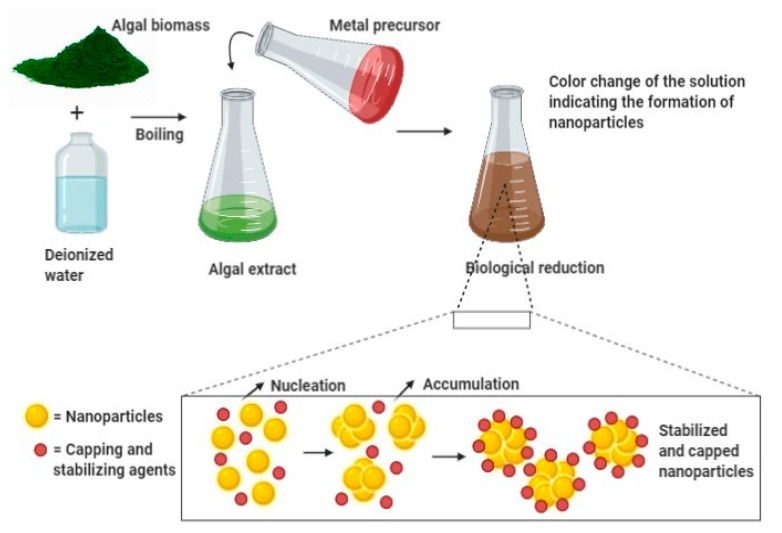
General mechanism for algal-mediated synthesis of inorganic/metallic nanoparticles.

Algal-mediated synthesis of inorganic NPs, such as Ag and Au, which are the most investigated noble metals to be used by the algal biomass for producing NPs, can be obtained by the three subsequent steps (Figure 1) [49]: (i) heating or boiling algal extract after mixing it with water or an organic solvent for a certain time period; (ii) preparation of molar ionic metallic solutions; (iii) exposure of the algal extract to the molar solution of the (noble) metal ion precursor in a flask under (or not) continuous stirring for a certain time period. It is worth noting that the final reaction results in a color change that determines the nucleation in which the adjacent nucleon particles bind together to form thermodynamically stable NPs of varying shapes and sizes [47]. For instance, Rajeshkumar et al. (2012) synthesized AgNPs by using aquatic brown alga, *Turbinaria conoides* (J.Agardh) [51]. The alga was first cleaned and made into fine powder. A specific amount of algal extract was then added into a metal precursor of AgNO_3_ solution and stored at ambient temperature under mechanical stirring. The change of color from brown to dark brown showed the formation of NPs, which displayed spherical morphology with a mean particle size of 96 nm.

Algal-mediated synthesis of inorganic NPs can either be achieved intracellularly or extracellularly [52]. In intracellular NPs synthesis, algal biomass is first collected and washed with distilled water. The biomass (live algae) is then treated with specific metallic solutions such as AgNO_3_ solution. The mixture is subsequently incubated at specific temperature, pH, and time conditions for bioreduction. It is eventually sonicated and centrifuged to yield the harvested stable NPs. In extracellular synthesis of NPs, algal biomass is first collected and washed with distilled water. Then, the following three methods are commonly used for the further procedure: (i) the algal biomass (dead algae) is dried under shadow for a specific time and the dried powder is treated with distilled water and filtered out; (ii) the algal biomass is sonicated with distilled water to obtain a cell-free extract; or (iii) the algal biomass is washed with distilled water and incubated for a few hours (8–16 h) and the obtained product is then filtered.

Besides, in addition to the phycosynthesis of metallic NPs via an enzyme (intracellular nitrogenase)-mediated route for the metal (e.g., Ag, Au) reduction in cyanobacteria (e.g., *Calothrix pulvinata*) [53], recent studies report an innovative method, namely ultrasound irradiation-assisted synthesis (UIAS) of AgNPs and AuNPs, using the cyanobacteria *Calothrix* (C.Agardh ex Bornet & Flahault) [54,55]. This eco-friendly and economical technique permits the acceleration of a wide range of chemical reactions and extraction procedures thanks to resulting cavitational collapses. Indeed, cavitational collapse produces intense local heating (~5000 °C) and high pressures (~2000 atmospheres) in the liquid reaction mixture, with noticeably short lifetimes [54,55].

In this illustration, algal biomass is first combined with deionized water and boiled at a certain temperature. The obtained algal extract is then treated with a metal precursor and incubated for a certain time at ambient temperature. The change of color indicates the formation of NPs. During the bioreduction of metal ions, the subsequent processes of nucleation and condensation (accumulation) assures the formation (growth) of stabilized NPs surrounded by capping agents. Then, several physical techniques can be used to characterize biosynthesized NPs. Thereby, the solution color change and SPR can be observed by UV-Vis spectroscopy, while the size, shape, and morphology of NPs can be determined by Dynamic Light Scattering (DLS) and/or Scanning Electron Microscopy (SEM) and/or TEM. In addition, their structure (functional groups) can be unraveled by FTIR spectroscopy, and their crystalline nature can be assessed by XRD spectroscopy.

### 5.1. Methods of Microalgal-Mediated AgNPs Synthesis 

Jena et al. (2013) reported the synthesis of AgNPs using the microalga *Chlorococcum humicola* (Nägeli) that belongs to the class *Chlorophyceae*, a freshwater, autotrophic and colonial microscopic organism that elicits high biomass productivity with high growth rate, while requiring minimum amount of sunlight and atmospheric CO_2_ for growth, making it very convenient and useful for pharmaceutical and nutraceutical purposes [56]. In this study, AgNPs were prepared by in-vivo and in-vitro synthesis by exposing the silver salt (AgNO_3_) solution at room temperature which caused the reduction of silver ion (Ag^+^). During the in-vitro synthesis, the formation of AgNPs was detected by a solution color change from translucent to dark reddish-yellow due to the excitation of SPR vibrations with the Ag^+^ whereas in in-vivo synthesis, the color change from bright green to dark brown is the indication of the formation of AgNPs. Both the in-vivo and in-vitro bioreduction synthesis systems confirm that AgNPs were randomly embedded in the cell surface of microalgae; they were evenly distributed throughout the algal biomass without agglomeration. The result from TEM analysis revealed that the AgNPs possessed a spherical shape with a crystalline structure and their sizes ranged from 2 to 16 nm with a mean size of 4 and 6 nm (Table 1).

More recently, Chokshi et al. (2016) investigated the biosynthesis of AgNPs using de-oiled biomass of *Acutodesmus dimorphus* (Turpin), a thermotolerant oleaginous microalga that has biofuel potential and displays antioxidant property [57]. This green alga is besides used as a biofertilizer and biostimulant [58]. In this study [57], the resulting AgNPs displayed antioxidant properties. The color change of the solution, from clear to brownish, indicated the development of AgNPs. The synthesized nanocrystals were of spherical morphology with a varying size from 2 to 15 nm as observed by atomic force microscopy (AFM) and TEM.

### 5.2. Methods of Macroalgal-Mediated AgNPs Synthesis 

Hashemi et al. (2015) reported the synthesis of AgNPs using the marine brown macroalga, *Padina boergesenii* (Allender & Kraft) [59]. This leafy rolled-blade alga exhibits a wide range of features including antidiabetic, antifungal, enzyme inhibition, anti-inflammatory and anti-angiogenic activities [60,61,62,63]. Due to the reduction of Ag^+^ in the algal solution and through a surface excitation plasmon vibration effect, the color of the solution turned into brownish yellow indicating the formation of AgNPs [59]. TEM micrographs demonstrated that AgNPs collected were crystalline in nature with spherical morphology and the particle size varied from 34.62 to 54.33 nm with the mean size of 43.3 nm (Table 1).

Similarly, Bhimba et al. (2015) investigated the biosynthesis of AgNPs using extract of *Gracilaria corticata* (J.Agardh) [64]. This red macroalga exerts potent activities including antioxidant, antifungal, and antibacterial properties and is applied in food colorants [65,66,67,68]. The bioreduction of AgNO_3_ solution resulted in a shift in color from clear to dark brown suggesting the development of AgNPs, which were spherical with particle sizes ranging from 10 to 35 nm as shown by TEM [64].

### 5.3. Methods of Cyanobacterial-Mediated AgNPs Synthesis

Mahdieh et al. (2012) showed extracellular formation of AgNPs by *Arthrospira* (formerly known as *Spirulina*) *platensis* (Gomont), a blue-green free-floating cyanobacterium mainly found in tropical and subtropical areas [69]. *S. platensis* is characterized mainly by cylindrical, multicellular trichomes, and displays heavy carbonate and bicarbonate ion concentrations [69]. This cyanobacterium is a potent protein source, animal feed supplement, and exerts antitumor, antioxidant and anti-inflammatory activities [70,71,72]. During the exposure of algal biomass, reduction of Ag^+^ ions occurred and consequent formation of AgNPs was indicated by a color change of solution from yellowish brown to dark brown [69]. TEM analysis showed that AgNPs were crystalline with a size ranging from 5 to 30 nm with a mean size of approximately 12 nm (Table 1).

Singh et al. (2014) performed the biosynthesis of AgNPs using the nitrogen-fixing cyanobacterium *Anabaena doliolum* (Bharadwaja) [73]. This filamentous freshwater species exerts antibacterial and antitumor activities and possesses unique features like salt tolerance and protection to nitrogenase activity. The bioreduction of Ag^+^ solution resulted in a shift in color from reddish blue to dark brown, indicating the formation of AgNPs) [73]. TEM micrographs showed well-dispersed spherical AgNPs with a particle size ranging between 10 and 50 nm [73,74].

### 5.4. Methods of Microalgal-Mediated AuNPs Synthesis

Shakibaie et al. (2010) reported the synthesis of AuNPs using *Tetraselmis suecica* (Kylin), a green marine microalga [81]. In this study, the algal extract reduced gold ions (Au^3+^ or Gold (III)) to AuNPs in the presence of chloroauric acid (HAuCl_4_). The resulting formation of AuNPs by this simple and cheap process was indicated by the color change of HAuCl_4_ solution from yellow to ruby red [81]. TEM analysis showed that the synthesized AuNPs were crystalline in nature, spherical, did not aggregate, and displayed an average particle size of 79 nm (Table 2).

Besides, Dahoumane et al. (2012) investigated the biosynthesis of AuNPs by the microalga *Klebsormidium flaccidum* (Kützing) [82]. This species possesses a unique feature of tolerating abiotic stresses such as cold acclimation, and can increase the osmotic concentrations of cells when submerged in hypertonic artificial water (e.g., after incorporating sucrose and amino acids) [82]. The color change of solution from yellow to purple, after bioreduction of Au^3+^, indicated the formation of AuNPs [82]. TEM analysis showed that the AuNPs were spherical and the size was about 9.0 ± 3.4 nm [82].

### 5.5. Methods of Macroalgal-Mediated AuNPs Synthesis

Singaravelu et al. (2007) reported the use of the brown macroalga, *Sargassum wightii* (Greville), for biosynthesis of AuNPs This seaweed is rich in sulfated polysaccharides and exerts a wide spectrum of biological properties such as free scavenging and antioxidant effects [83]. Biosynthesis process of AuNPs implicated the use of HAuCl_4_ and a change in color from yellow to ruby red after reduction of Au^3+^ [83]. TEM micrographs showed monodispersed AuNPs, ranging from 8 to 12 nm, with an average size of 11 nm (Table 2).

Singh et al. (2012) investigated the synthesis of AuNPs using the brown seaweed *Padina gymnospora* (Kützing) [84]. This macroalgae is known for its potent antioxidant, antibacterial and wound healing properties [84]. During the AuNPs synthesis, the bioreduction of Au^3+^ occurred in the presence of the algal biomass, and this was confirmed by the color change of the solution from yellow to ruby red [84]. SEM analysis showed that the synthesized AuNPs were spherical-shaped with a particle size between 53 and 67 nm [84].

Venkatesan et al. (2014) reported biosynthesis of AuNPs by reduction of Au^3+^ using the novel edible marine brown macroalga *Ecklonia cava* (Kjellman) [85]. Synthesized AuNPs showed good antimicrobial and biocompatibility with human keratinocyte cell line [85]. Interestingly, the reaction was completed within 1 min at 80 °C [85]. Further, FTIR spectroscopy revealed that AuNPs were functionalized with biomolecules that have primary amine group, hydroxyl group and other stabilizing functional groups [85]. Additionally, XRD pattern showed high purity and face-centered cubic structure of the AuNPs [85]. Eventually, microscopy results showed that these AuNPs were formed with shapes (e.g., spherical and triangular) with an average size of 30 ± 0.25 nm [85].

### 5.6. Methods of Cyanobacterial-Mediated AuNPs Synthesis 

Kalabegishvili et al. (2012) reported the extracellular synthesis of AuNPs using the blue-green alga, *S. platensis* (Gomont) [86]. As previously described, this multicellular filamentous helical cyanobacterium is often used as a medicinal matrix and food additive [86]. Interestingly, the biosynthesis process of AuNPs from *S. platensis* is simple, rapid, economical, and involves bioreduction of Au^3+^. The resulting AuNPs were mostly of spherical morphology, some of the NPs also showed face-centered cubic structures, and their size ranged from 15 to 60 nm with an average size of 30 nm, as seen by TEM analysis (Table 2).

Lenartowicz et al. (2017) investigated the biosynthesis AuNPs with various shapes using the filamentous cyanobacterium, *Anabaena laxa* (Rabenhorst) [87]. The reaction was performed by taking low concentrations of HAuCl_4_ to avoid death of this sensitive cyanobacterium [87]. After bioreduction of Au^3+^, the color of the solution changed from transparent to purple indicating the formation of AuNPs [87]. The TEM analyses of the obtained AuNPs revealed variable morphology (e.g., spherical, triangular, hexagonal, and irregular shape), and the particles size mostly ranged between 0 and 30 nm although few larger NPs ranged in size between 30 and 100 nm [87].

Kumar et al. (2016) demonstrated the accelerated, high product yield, and cost-effective synthesis of AuNPs by the simple UIAS in an ecofriendly manner using the *cyanophyceae Calothrix* [54]. The appearance of a light pink color at *λ*_max_ = 550 nm indicated the synthesis of AuNPs [54]. The XRD spectrum of the AuNPs exhibited a Bragg reflections peak at 38.23, corresponding to the elemental gold [54]. TEM images showed the formation of anisotropic AuNPs with predominant truncated shape and particles are in the range of 30–120 nm [54]. Further, AuNPs showed significant catalytic efficiency in reducing 4-nitrophenol to 4-aminophenol, proving its potential usefulness in remediation of toxic chemicals and other catalyst based industrial applications [54].

### 5.7. Algal-Mediated Synthesis of Other Types of Nanoparticles 

**Figure 2:**
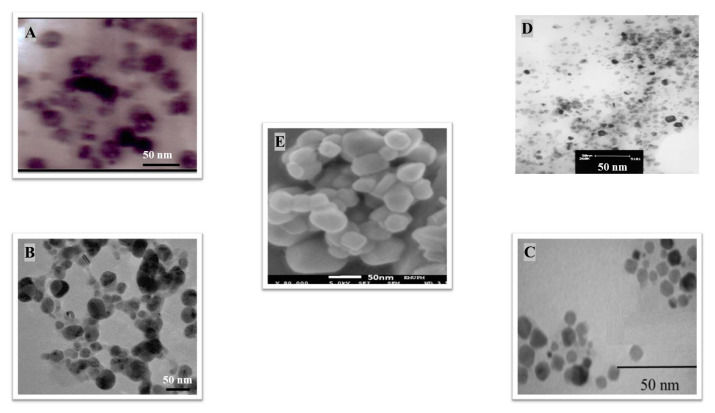
TEM images of various metallic nanoparticles (NPs). (**A**) Cadmium sulfide NPs (CdSNPs) by *Phormidium tenue* [90], (**B**) copper oxide NPs (CuONPs) by *Bifurcaria bifurcata* [92], (**C**) iron oxide NPs (Fe_2_O_3_NPs) by *Sargassum muticum* [94], (**D**) palladium NPs (PdNPs) by *Spirulina platensis* [97], (**E**) zinc oxide NPs (ZnONPs) by *Sargassum muticum* [99]. (Copyright permission has been granted for each figure).

The algal-mediated synthesis of metallic NPs other than AgNPs and AuNPs is also possible by reduction of metal ions (Ag^+^ or Au^+^) [53]. Indeed, different NPs such as cadmium sulfide (CdS), CuO, iron oxide (Fe_2_O_3_), palladium (Pd) and ZnONPs can be prepared either from microalgae, macroalgae or cyanobacteria (Figure 2A–E).

Thereby, CdSNPs could be synthetized by using the cyanobacterium *Phormidium tenue* (Gomont), which is known to exhibit special photochemical and photophysical properties [90]. The bioreduction reaction of cadmium ions (Cd^2+^) was monitored until the color changed from yellow to orange, which indicated the formation of CdSNPs [90]. TEM analysis showed that these NPs were spherically shaped with a mean particle size of 5 nm (Figure 2A).

Another study led by Mandal et al. (2016) showed the algal-mediated synthesis of CdSNPs using *S. platensis* (Gomont) that was initially cultured in the laboratory at ambient temperature [91]. After harvesting and shade drying in petri plates, the pellets were homogenized with liquid N_2_ in an autoclaved mortar for extraction. The obtained mixture was then centrifuged for 20 min at 4 °C and the supernatant was stored for further analysis. Cadmium nitrate (Cd(NO_3_)_2_) solution was added to the algal mixture for the synthesis of CdSNPs and yellow color appearance confirmed the formation of CdSNPs [91]. The TEM image showed that the synthesis was intracellular and the NPs were spherical in shape with an average diameter of 8–12 nm [91].

Besides, CuONPs could be prepared using an extract of the brown macroalga, *Bifurcaria bifurcate* (R.Ross), which is known to exert antimicrobial, antioxidant and antitumoral activities [92]. The biosynthesized CuONPs were observed by a color change, classically due to the SPR phenomenon, from deep blue to dark red, after bioreduction of copper ions (Cu^2+^) with water-soluble diterpenoids abundantly present in the algal extract [92]. From TEM micrographs, CuONPs exhibited a spherical morphology and a particle size ranging from 5 to 45 nm, with a mean size of 20.66 nm (Figure 2B). 

Another study led by Bhattacharya et al. (2019) reported the formation of CuONPs by using *Anabaena cylindrica* (Lemmermann) [93]. This filamentous photoautotrophic cyanobacterium is capable of fixing nitrogen [93]. The synthesized CuONPs were used as disinfectant for purifying drinking water and as an antimicrobial against pathogen strains of *Escherichia coli* [93]. These NPs were crystalline in nature as revealed by XRD and SEM pattern analyses [93]. Their average particle size was of 3.6 nm as measured by a zetasizer instrument [93].

Moreover, Fe_3_O_4_NPs have tremendous potential in many applications such as biosensors, environmental remediation, magnetic storage media, ferrofluids, and catalysts [94]. Indeed, Fe_3_O_4_NPs possesses a cubic inverse spinel structure that exhibit unique electric and magnetic properties [94]. Interestingly, these NPs were synthesized by Mahdavi et al. (2013) using an aqueous extract of the invasive/fast-growth-rate seaweed *Sargassum muticum* (Yendo), commonly known as Japanese wireweed [94]. During the biosynthesis reaction and after the bioreduction of ferric ions (Fe^3+^) using ferric chloride (FeCl_3_) as a metal precursor, the rapid color change of the solution from yellow to dark brown indicated the formation of Fe_3_O_4_NPs [94]. TEM analysis showed that these NPs were cubic and presented a particle mean size of 18 ± 4 nm (Figure 2C).

Another study led by El-Kassas et al. (2016) reported the synthesis of Fe_3_O_4_NPs using two brown seeweeds: *Padina pavonica* (Linnaeus) and *Sargassum acinarium* (Linnaeus) [95]. The samples were washed to remove contaminants, dried at −20 °C and then ground to powdered form. Fe_3_O_4_NPs were then prepared by co-precipitation method [95]. Briefly, FeCl_3_ solution was added to the algal extract and NPs were obtained by reduction process. The mixture was then stirred and centrifuged. The color change from yellow to brown indicated the synthesis of Fe_3_O_4_NPs. TEM image showed spherical NPs in size range of 10–19.5 nm for *P. pavonica* and 21.6–27.4 nm for *S. acinarium* [95].

Furthermore, PdNPs were made by Momeni et al. (2015) using the brown marine macroalga *Sargassum bovinum* (C.Agardh), which is known to display electrocatalytic activities towards H_2_O_2_ [96]. Due to the presence of water-soluble compounds in the algal extract, the reduction of palladium ions (Pd^2+^) to PdNPs occurred with color changes in the solution from yellow to dark brown [96]. The resulting synthesized NPs displayed a mean particle size of 5–10 nm with octahedral morphology as shown by TEM analysis (Figure 2D).

In addition, Sayadi et al. (2018) investigated the biosynthesis of PdNPs by the filamentous Gram-negative cyanobacterium *S. platensis* (Gomont) [97]. After bioreduction of Pd^2+^, the color of the solution changed from yellow to dark brown, indicating the formation of PdNPs) [97]. The obtained NPs were spherical in shape with a particle size ranging from 10 to 20 nm, as observed by TEM [97].

Eventually, Priyadharshini et al. (2014) investigated the extracellular synthesis of ZnONPs using extracts from the red macroalga, *Gracilaria edulis* (S.G.Gmelin) [98]. This agarophyte exerts a wide variety of activities including anti-viral, anti-cancer, anti-bacterial and anti-fungal [98]. After bioreduction of zin ions (Zn^2+^) using zinc nitrate (Zn(NO_3_)_2_) as metal precursor, a change of color from white to pale brown was observed, indicating the formation of ZnONPs [98]. The obtained NPs were rod-shaped with a particle size ranging from 66 to 95 nm [98].

Another study published in the same year by Azizi et al. (2014) reported such biosynthesis using an aqueous extract of the brown macroalga *Sargassum muticum* (Yendo), previously evoked for the biosynthesis of Fe_2_O_3_NPs [99]. After bioreduction of Zn^2+^, a change of color from dark brown to pale white, indicating the formation of ZnONPs [99]. These NPs were hexagonal shapes with a particle size ranging between 30 and 57 nm as noticed from SEM analysis (Figure 2E).

## 6. Major Bioapplications and Underlying Molecular Mechanisms of Algal-Mediated Synthesis of Metallic NPs

**Figure 3:**
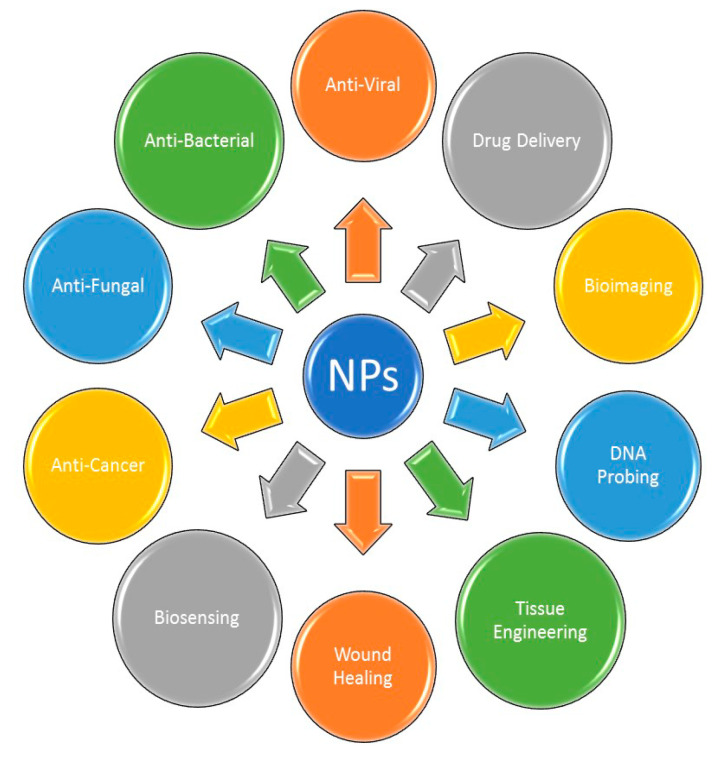
Major applications of metallic NPs.

There are several applications reported for the algal-mediated synthesis of inorganic NPs. Metallic NPs and NPs, such as AgNPs and AuNPs, have been broadly considered for use in applications in a different scope of biotechnological, medical, cosmetical and pharmaceutical fields. The main applications of these NPs in the fields of medicine and biotechnology are illustrated in Figure 3.

**Figure 4:**
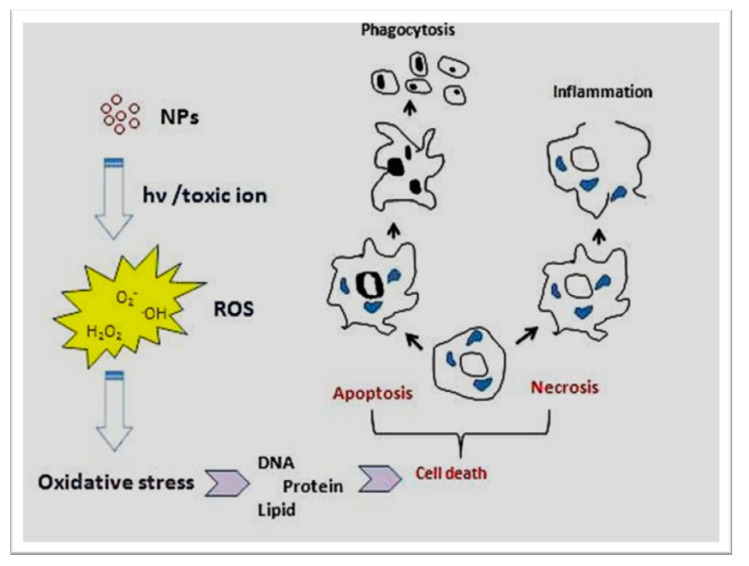
Major molecular and cellular mechanisms involved in the toxicity of (metallic) NPs [100]. (Reproduced with permission).

The physicochemical reactivity of (metallic) NPs leads to oxidative stress due to the resulting formation of free radicals or ROS (e.g., superoxide radical anions and hydroxyl radicals) directly or indirectly (i.e., through activation of oxidative enzymatic pathways), as shown in Figure 4. Some metal oxide NPs, such as titanium dioxide NPs (TiO_2_NPs) used in cosmetics, food additives and cancer therapy, are water-insoluble but would induce enhanced toxicity effects (e.g., oxidative stress, phototoxicity, genotoxicity and immunotoxicity), especially at concentrations higher than 100 μg/mL [100]. Despite other NPs such as ZnO, TiO_2_NPs do not release toxic ions hence toxicity of these NPs (i.e., ROS, mitochondrial depolarization, plasma membrane leakage, intracellular calcium influx and cytokine release) could be attributed to their size-dependent interaction/adsorption with intracellular biomolecules [100]. Additionally, it was shown that phototoxicity of these NPs could be decreased via surface coating with CS because of the prevention of biomolecule adsorption and hydroxyl radicals (·OH) production in the photoactivation process [100].

### 6.1. Some Medical Applications and Underlying Molecular Mechanisms of Algal-Mediated Synthetized AgNPs

AgNPs possess good conductivity and are overly sensitive towards surface absorption of the metals. Thereby, AgNPs are often associated with many useful theranostic applications including biosensing, imaging, drug delivery, wound healing, cancer and microbial (e.g., bacteria, some fungi) therapies [2]. Due to the efficient antimicrobial properties, AgNPs are used in a broad spectrum of consumer products such as cosmetics, electronics, textiles, and food products [100]. 

For instance, topical ointments (e.g., creams) or implants impregnated with Ag polymers were conceived to avoid infections of burned and damaged areas, because AgNPs are known to cause cell membrane lysis of bacteria [2]. Furthermore, biosynthesized NPs can be used as efficient controlled and disease targeted drug-delivery systems if they are able to escape the immune system by overcoming biological barriers and the complex tumor microenvironment (TME) [101].

Interestingly, Khalid et al. (2017) reported anti-bacterial, anti-fungal, anti-cancerous and antiviral properties of AgNPs synthesized by ethanolic extract of three freshwater microalgae strains, namely HM1 (DHM1), HM2 (DHM2), both derived from *Dictyosphaerium* sp. (Nägeli), and HM3 (PHM3) from *Pectinodesmus* sp. (Meyen) [102]. Significant activity of these AgNPs was reported against 14 bacterial strains, the fungal strain *Candida albicans*, the hepatocellular carcinoma (HepG2) and breast cancer (MCF7) cell lines and against the Newcastle Disease Virus (NDV) on Huh7-infected cells [102]. Unfortunately, as an example of several studies, the underlying molecular mechanisms have not been mentioned in this published work. 

Besides, Venkatesan et al. (2016) demonstrated that AgNPs, synthesized by extracts from brown seaweed *Ecklonia cava* (Kjellman), elicit a significant anti-bacterial activity against *Escherichia coli* and *Staphylococcus aureus*, as well as an efficient antioxidant activity in vitro, and an anti-cancer activity against human cervical (HeLa) cells by a mechanism involving apoptosis [103].

Considering the wide use of AgNPs in medicine, toxicity assay is an important factor to keep in mind. Cytotoxicity of these NPs, independently of their surface coating, is related to comfortable oxidation AgNPs to Ag^+^ ions which are very toxic for biological systems and cellular components (e.g., DNA) [100]. It is postulated that AgNPs in an aqueous system are more toxic compared to the bulk. Ag is more toxic due to the presence of dissolved oxygen, its reduction on NPs and then the release of H_2_O_2_ from AgNPs [100]. Additionally, results demonstrated that ROS generation from AgNPs are greater than that of macro (bulk) silver, and those AgNPs with higher intracellular Ag release are also more toxic [100].

### 6.2. Some Medical Applications and Underlying Molecular Mechanisms of Algal-Mediated Synthetized Gold Nanoparticles

AuNPs are one of the most promising inorganic NPs that have attracted scientific and technological interests due to their ease of synthesis, chemical stability, and excellent optical and electronic properties [100]. These unique properties make them appealing tools for biomedical applications in radiology as radiation enhancer, in targeted-drug delivery, in biosensing for biomolecular hypersensitive detection (biosensing), in cancer diagnostics and cancer therapy by hyperthermal treatment [104]. 

The application of AuNPs in such fields depends primarily on the capacity to synthesize particles with regulated shape, monodispersity, size, stability, and chemical composition. Interestingly, they can be easily prepared by various methods (i.e., physical, chemical and the growing safe and effective biological approach) due to their exceedingly small diameter, and thereby can be easily transferred to tissues and cells just like DNA and proteins [105].

For instance, Murugesan et al. (2015) showed that the AuNPs, synthesized by the red macroalgae, *Hypnea musciformis* (Wulfen), which besides is known as a highly opportunistic invader causing large floating blooms, are able to exert anti-fungal properties against *Aspergillus niger* and *Mucor* spp. [106].

Whereas AgNPs have attracted much interest as antimicrobials, the development of AuNPs has largely contributed to a new field of research, namely cancer nanomedicine. This is because, in comparison to traditional anti-cancer drugs, NPs provide a targeted approach which prevents undesirable effects [105,107]. Thereby, González-Ballesteros et al. (2017) performed the synthesis of AuNPs using a brown alga *Cystoseira baccata* (S.G.Gmelin) [89]. The alga was washed with distilled water and the fragments were converted into fine pieces. The solution was then boiled and centrifuged at 4500 rpm for 10 min. Aqueous solution of HAuCl4 was added to the algal extract and kept at room temperature with stirring at regular intervals. The TEM analysis showed the formation of spherical gold nanoparticles with an average particle size of 8.4 ± 2.2 nm (Table 2). The synthesized AuNPs were then tested against human colon cancer Caco-2 and HT-29 cells for which they showed a significant anticancer effect [89].

Although most of in vitro investigations have demonstrated that AuNPs are non-toxic for cells, their cytotoxicity depends on their uptake and intracellular distribution which themselves rely on the size and shape of AuNPs as well as on their surrounding ligands [100]. For instance, the cytotoxicity effects, observed with Balb/3T3 mouse fibroblasts treated with 5-nm AuNPs but not with 15-nm AuNPs, were explained by the high number of small AuNPs taken-up by cells in comparison to the larger particle [108]. It is stated that anisotropic AuNPs (e.g., (nanorods, nanourchins and nanocages) have more potential oxidation than the isotropic ones due to their highly exposed surface areas and defects [109]. In addition, some studies indicated that spherical AuNPs are more suitable for biomedical applications [100].

## 7. Conclusions and Perspectives

In this article, we highlighted the biological method of synthesis of metallic NPs using algae. A special emphasis was given to AgNPs and AuNPs because of their unique features that have attracted much scientific and technologic interests. We described the main factors influencing a stable and efficient biosynthesis, as well as the most used algae for such purposes. We aimed to demonstrate the production of different sized NPs with many desirable shapes using various species of algae. We also provide some applications of metallic NPs in the field of nanomedicine, and their underlying main molecular mechanism-induced cytotoxicity. It is worth noting that algal-mediated NPs synthesis is a fast, cost-effective, and efficient strategy that has opened a way for nanotechnologists to produce desirable nanomaterials with clean energy processes. Nevertheless, there is a need to explore more in this field before these eco-friendly NPs are safely translated in medicine.

## Figures and Tables

**Table 1:** Algae-mediated synthesis of silver nanoparticles (AgNPs). (Copyright permission has been granted for each figure).

Algae	Algal Morphology	Morphology	Nanoparticles	Size (nm)	Reference
*Chlorococcum humicola* (Nägeli) **Microalgae**	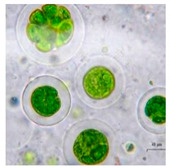	Spherical	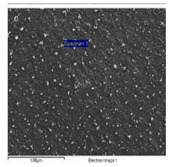	2–6	[56]
*Chlorella vulgaris* (Beyerinck) **Microalgae**	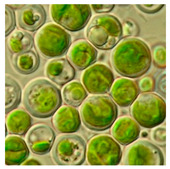	Spherical	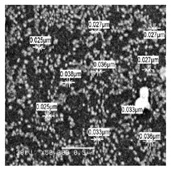	37–59 to 163–205	[75]
*Botryococcus braunii* (Kützing) **Microalgae**	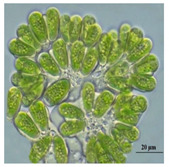	Cubical, spherical, truncated, triangular	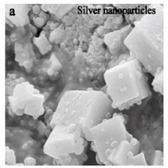	40–100	[76]
*Sargassum angustifolium* (C.Agardh) **Macroalgae**	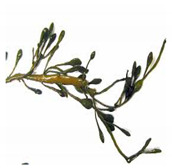	Spherical	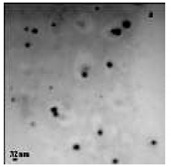	22–42	[36]
*Sargassum muticum* (Yendo) **Macroalgae**	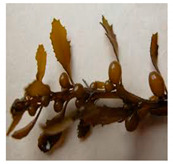	Spherical	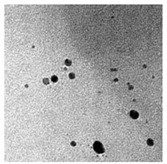	5–15	[77]
*Cystophora moniliformis* (Esper) **Macroalgae**	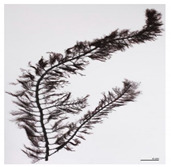	Spherical, polydispersed	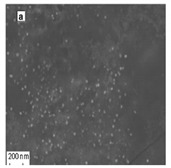	81	[78]
*Ulva fasciata* (Delile) **Macroalgae**	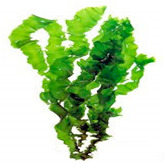	Crystalline	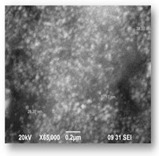	33	[79]
*Pithophora oedogonia* (Mont.) **Macroalgae**	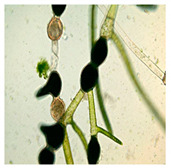	Cubical, sometime hexagonal	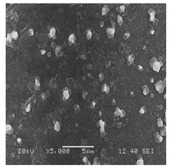	25–44	[80]
*Padina boergesenii* (Allender & Kraft) **Macroalgae**	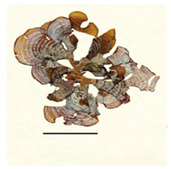	Spherical	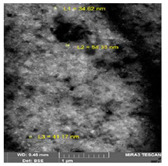	34–54	[59]
*Spirulina platensis* (Gomont) *Cyanobacterium*	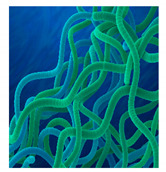	Cubic	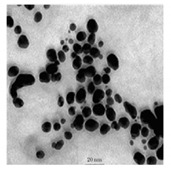	12	[69]

**Table 2:** Algal-mediated synthesis of gold nanoparticles (AuNPs). (Copyright permission has been granted for each figure).

Algae	Algal Morphology	Morphology	Nanoparticles	Size (nm)	Reference
*Tetraselmis suecica* (Kylin) **Microalgae**	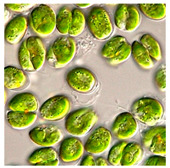	Spherical	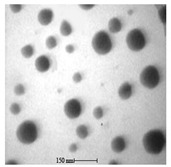	79	[81]
*Sargassum wightii* (Greville) **Macroalgae**	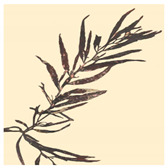	Thin planner	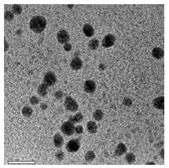	8–12	[83]
*Turbinaria conoides* (J.Agardh) **Macroalgae**	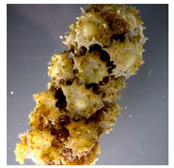	Spherical, triangle, sometime pseudo-spherical	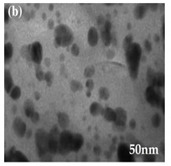	6–10	[88]
*Cystoseira baccata* (S.G.Gmelin) **Macroalgae**	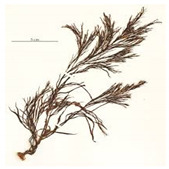	Spherical	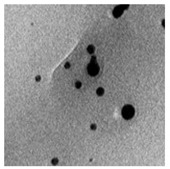	8.4 ± 2.2	[89]
*Spirulina platensis* (Gomont) *Cyanobacterium*	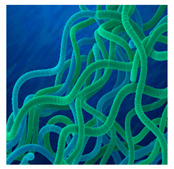	Spherical, cubic	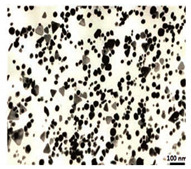	15–60	[86]

## Abbreviations

Silver	Ag
Atomic force microscopy	AFM
Gold	Au
Cadmium sulfide	CdS
Chitosan	Cs
Copper (II) oxide	CuO
Tumor microenvironment	TME
Dynamic light scattering	DLS
Iron (II) chloride	FeCl_2_
Iron (III) chloride	FeCl_3_
Fourier-transform infrared	FTIR
Chloroauric acid	HAuCl_4_
Magnetic resonance imaging	MRI
Nanoparticles	NPs
Palladium	Pd
Polyunsaturated fatty acids	PUFAs
Reactive Oxygen Species	ROS
Scanning electron microscopy	SEM
Surface plasmon resonance	SPR
Titanium dioxide	TiO_2_
Transmission electron microscopy	TEM
Ultrasound irradiation-assisted synthesis	UIAS
Ultraviolet-Visible	UV-Vis
X-ray powder diffraction	XRD
Zinc oxide	ZnO
